# Regulation of redox homeostasis in cadmium stressed rice field cyanobacteria by exogenous hydrogen peroxide and nitric oxide

**DOI:** 10.1038/s41598-021-82397-9

**Published:** 2021-02-03

**Authors:** Nidhi Verma, Sheo Mohan Prasad

**Affiliations:** Ranjan Plant Physiology and Biochemistry Laboratory, Department of Botany, University of Allahabad, Prayagraj, 211002 India

**Keywords:** Plant sciences, Plant signalling, Plant stress responses

## Abstract

In the present study, defensive strategies of H_2_O_2_ mediated NO signaling were analyzed in Cd stressed *Nostoc muscorum* and *Anabaena* sp. Exogenously supplied SNP (10 µM) and H_2_O_2_ (1 µM) lessen the toxicity of Cd (6 µM) but without NO; H_2_O_2_ was unable to release the stress from cyanobacterial cells potentially. The reduced contents of exopolysaccharide, protein content, endogenous NO and enzymatic antioxidants (SOD, POD, CAT, and GST) due to Cd toxicity, were found increased significantly after exogenous application of H_2_O_2_ and SNP thereafter, cyanobacterial calls flourished much better after releasing toxic level of Cd. Moreover, increased level of ROS due to Cd stress also normalized under exogenous application of H_2_O_2_ and SNP. However, chelation of NO hindered the signaling mechanism of H_2_O_2_ that diminished its potential against Cd stress while signaling of NO has not been hindered by chelation of H_2_O_2_ and NO potentially released the Cd stress from cyanobacterial cells. In conclusion, current findings demonstrated the synergistic signaling between H_2_O_2_ and NO towards the improvement of cyanobacterial tolerance to Cd stress, thereby enhancing the growth and antioxidant defense system of test cyanobacteria that improved fertility and productivity of soil even under the situation of metal contamination.

## Introduction

Cadmium (Cd) contamination in agricultural fields like rice crop fields has become a serious environmental issue. Potentially active Cd ions in the soil can accumulate in rice plants and ultimately reduce the uptake and translocation of vital minerals and nutrients for plants^1–3^. Cd ions in agricultural soil can also negatively affect the beneficial microbes present in the rice field^4^. Cyanobacteria are pioneer phototrophic prokaryotic microorganisms that are known to display their potential role in sustainable agricultural development especially for paddy fields. *Nostoc muscorum* and *Anabaena* sp. are categorized as heterocystous cyanobacteria and known for their nitrogen-fixing ability. They are mainly present on soil and water bodies and fix about 20–25 kg/ha atmospheric nitrogen. More efficiently, *Anabaena* sp. can fix about 60 kg/ha of nitrogen in a season^5^. Unfortunately, Cd ions present in rice soil are major threats to these nitrogen fixers. Previous studies demonstrated that some photosynthetic bacteria can tolerate up to 1 mg Cd kg^−1^ soil in the association with Hay bacillus (*Bacillus subtilis*) and some other lactic acid bacteria^6^. But the release of Cd is much higher (200 mg Cd kg^−1^ soil) in the soils near the leather and electro-plating factories^7^ that creates a hazardous environment for paddy field cyanobacteria which ultimately results to the poor quality of rice crop. Cd interrupts the cell functioning and hangovers the regular metabolic processes of cyanobacteria. Thus, Cd ions can enter into the cell through P-type Cd^2+^ ATPases (e.g. *cadA1* and *cadA2*) and interrupt electron transport chain and produce excessive ROS (reactive oxygen species thereby disturbing the balance between antioxidants and ROS which tend to the cell death^8^. Study of Qiao et al.^9^ shows that there are three possible ways through which Cd imposes ROS accumulation in cells: (1) excess level of Cd^2+^ enhances the expression of miR398 that ultimately inhibits the functioning of Cu/Zn/SOD and finally, a drastic rise of ROS; (2) excess Cd^2+^ in cells can inhibit the regulatory role of second messengers like Ca^2+^, or other signaling molecules like NO and H_2_O_2_; (3) excess accumulation of Cd^2+^ enhances the activity of NADPH oxidase, that is a major producer of ROS. So to enhance the tolerance against different environmental stresses the external applications of different signaling molecules like H_2_O_2_, NO, and H_2_S, are considered as one of the most beneficial methods^10^.

NO and H_2_O_2_ are the potentially active and key biological signaling molecules known for the plant defense. At a low and very specific concentration, H_2_O_2_ and NO work as signal messengers for cell communication during stress situations. Tiwari et al*.*^11^ reported that exogenous supplementation of SNP (a donor of NO) reduced the level of ROS which is produced by aluminium stress in *Anabaena* PCC 7120. Verma et al*.*^12^ reported that in stress situation the increased ROS (O_2_^•−^, H_2_O_2_, ^•^OH, ^1^O_2_) level promotes the production of reactive nitrogen species (RNS: NO, ONOO^−^, N_2_O_3_, NO_2_) that finally control the balancing of antioxidants to protect the plants from severe damage. Several recent findings show the existence of several signaling topologies between NO and H_2_O_2_^13^. It has been reported that H_2_O_2_ mediates some other signaling molecules like NO or H_2_S to regulate oxidative stress^14^. Hasanuzzaman et al*.*^15^ suggested that Cd toxicity has been removed expressively by pretreatment of H_2_O_2_ in the seedlings of *Brassica nupus*. Pretreatment of H_2_O_2_ possibly empowers the internal defense system of the organism, but its downstream signal transduction mechanisms and pathways are not effusively clear in cyanobacteria. The defense strategies in organisms are a much complex process in which different signaling molecules are linked together as reported in the study of Li et al*.*^16^; it is proven that there is a multifaceted interaction among NO, H_2_S, and H_2_O_2_ promoting thermotolerance in maize seedlings. Christou et al*.*^17^ also noticed a faster and stronger response against salt stress by the priming of H_2_O_2_ and SNP (donor of NO) in strawberry plants. Gonzalez et al*.*^18^ have also revealed the cross linking mechanism of calcium, H_2_O_2_, and NO in copper treated *Ulva compressa*. However, in the case of cyanobacteria to the best of our knowledge the interacting role of signaling molecules H_2_O_2_ and NO in Cd stress alleviation is lacking. Therefore, we have hypothesized that synergistically H_2_O_2_ and NO could initiate certain protective mechanisms that may be associated with metal stress particularly Cd tolerance in cyanobacteria.

Hence, the prime focus of this study was to evaluate the potential role of H_2_O_2_ and NO as defense signaling molecules to cadmium stress tolerance in cyanobacteria *Nostoc muscorum* ATCC 27893 and *Anabaena* sp. PCC 7120. Also a key objective was to explore the probable mechanism and connective pathway between H_2_O_2_ and NO.

## Results

### Effect of H_2_O_2_ and NO on growth of Cd stressed cyanobacteria

**Figure 1:**
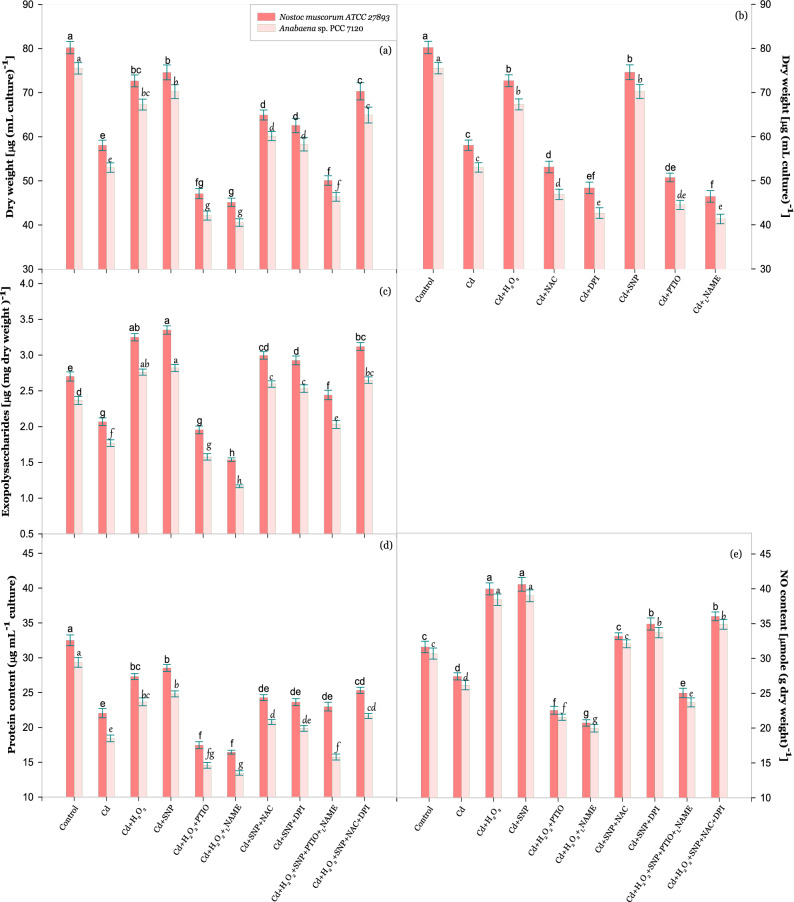
Effect of H_2_O_2_ and NO (SNP) on growth **(a,b)** and contents of exopolysaccharides **(c)**, protein **(d)** and endogenous NO **(e)** of *Nostoc muscorum* ATCC 27893 and *Anabaena* sp. PCC 7120 exposed to Cd after 24 h of treatment. Data presented are means ± standard error of three independent experiments with three replicates in each experiment (n = 9). Bars with different letters represent significant difference at P < 0.05 significance level according to the DMRT.

In the present study growth of both the tested cyanobacteria was measured in terms of dry weight as shown in Fig. 1a,b which depicted that 6 µM Cd inhibited the growth by 28% in *Nostoc muscorum* ATCC 27893 and 30% in *Anabaena* sp. PCC 7120 (hereafter referred as *Nostoc muscorum* and *Anabaena* sp.) in comparison to control. However, on exogenous supplementation of H_2_O_2_, growth was recovered and inhibition was noticed only 9 and 11% respectively for *Nostoc muscorum* and *Anabaena* sp. Similar to this, exogenous SNP significantly reduced (P < 0.05) the inhibition exerted by Cd, and found only 11 and 7%, respectively as compared to control. Further, to know the regulatory role of endogenous ROS and NO on growth, Cd treated cells were subjected to NAC, DPI, PTIO and _L_NAME; therefore, a greater reduction in dry weight was noticed under PTIO and _L_NAME treatment in comparison to control (Fig. 1b). Furthermore, to clarify the cross-talk of H_2_O_2_ and NO, cells were treated with scavengers (NAC and PTIO, respective scavengers of H_2_O_2_ and NO) and inhibitors (DPI and _L_NAME, respective inhibitors of NADPH oxidase and nitric oxide synthase (NOS) enzymes) with Cd stress. In this case, our results showed a critical decline in growth under treatments of PTIO or _L_NAME i.e. 41 and 44% respectively in *Nostoc muscorum* and 44 and 46% respectively in *Anabaena* sp. even in the presence of H_2_O_2_. Contrastingly, growth was found improved moderately under SNP treatment even in the presence of NAC and DPI (Fig. 1a). Moreover, in a combined treatment of both the signaling molecules, H_2_O_2_ and SNP along with both PTIO and _L_NAME under the same stress condition, inhibition in growth again found crucial i.e. 38 and 39% in *Nostoc muscorum* and *Anabaena* sp. respectively. Whereas, under similar stress, combined treatment of H_2_O_2_ and SNP along with NAC and DPI inhibition in growth was reduced and found only 12 and 14% in *Nostoc muscorum* and *Anabaena* sp. respectively on comparison to control.

### Effect of H_2_O_2_ and NO on the secretion of exopolysaccharide (EPS) layer during Cd stress

Impact of exogenous supplementation of H_2_O_2_ and SNP on secretion of defensive layer of EPS in test cyanobacteria has been portrayed in Fig. 1c. In both the test cyanobacteria *Nostoc muscorum* ATCC 27893 and *Anabaena* sp. PCC 7120 secretion of the EPS layer was declined significantly (P < 0.05) by 23 and 25% respectively under Cd treatment in contrast to control values (Fig. 1c). Under the treatment of Cd + H_2_O_2_ and Cd + SNP, the EPS secretion was enhanced significantly (P < 0.05) by 20 and 24% respectively in *Nostoc muscorum* and 17 and 19% respectively in *Anabaena* sp. comparative to control values. Contrastingly, under the same stress, the decline in EPS secretion was found more critical under the treatments of PTIO or _L_NAME i.e. 28 and 43% respectively in *Nostoc muscorum* and 33 and 51% respectively in *Anabaena* sp. even in the presence of H_2_O_2_. Whereas, treatments of NAC or DPI along with SNP do not hinder the signaling mechanism of NO and an increased EPS content was noticed i.e. 11 and 8% respectively in *Nostoc muscorum* and 10 and 7% respectively in *Anabaena* sp. Moreover, combined treatments of H_2_O_2_ and SNP along with PTIO and _L_NAME under the same stress crucially declined the EPS content that showed the incapability of H_2_O_2_ without NO. On the other hand, enhanced EPS content was noticed on the combined treatment of H_2_O_2_ and SNP along with NAC and DPI under the same stress showed the potential of NO without H_2_O_2_ in the removal of the toxic impact of Cd on EPS secretion.

### Effect of H_2_O_2_ and NO on protein content in Cd stressed cyanobacteria

The results pertaining to the effect of H_2_O_2_ and SNP supplementation on the protein content in Cd challenged *N. muscorum* and *Anabaena* sp. have been depicted in Fig. 1d. Cd at 6 µM doses significantly decreased the protein content by 32% in *N. muscorum*, and by 37% in *Anabaena* sp. respectively, in comparison to control values. However, exogenous supplementation of H_2_O_2_ and SNP to Cd stressed cyanobacteria, considerably lowered the inhibitory effect of Cd on protein content but values were still less than control. Contrastingly, all the positive effects of H_2_O_2_ and SNP on protein content were reversed on the application of PTIO and _L_NAME. Whereas application of NAC and DPI does not alter the role of SNP on protein content.

### Effect of exogenous H_2_O_2_ and NO on the content of endogenous NO during Cd stress

The impact of exogenously supplied H_2_O_2_ and SNP on the endogenous level of NO have been depicted in Fig. 1e. The 6 µM Cd reduced the NO accumulation significantly (P < 0.05) by 13 and 15% in *Nostoc muscorum* and *Anabaena* sp. respectively. Contrastingly, exogenously supplied H_2_O_2_ significantly (P < 0.05) enhanced the content of endogenous NO by 26 and 25% and more pronouncedly SNP enhanced (P < 0.05) the NO content inside the cell by 28 and 27% in *Nostoc muscorum* and *Anabaena* sp. respectively. Under similar stress, a very severe reduction in NO content was noticed on the separate treatments of PTIO and _L_NAME even in the presence of H_2_O_2_. In contrary to this exogenous supplementation of SNP slightly improved NO content even in the presence of NAC and DPI (Fig. 1e). Moreover, under the same stress, combined treatment of H_2_O_2_ and SNP along with PTIO and _L_NAME critically declined the NO content by 21 and 23% in *Nostoc muscorum* and *Anabaena* sp. respectively while endogenous NO content was found enhanced by 14 and 13% in *Nostoc muscorum* and *Anabaena* sp. respectively on combined exposure of H_2_O_2_ and SNP along with NAC and DPI under the same stress.

### Effect of H_2_O_2_ and NO on intracellular Cd accumulation under Cd stress

**Figure 2:**
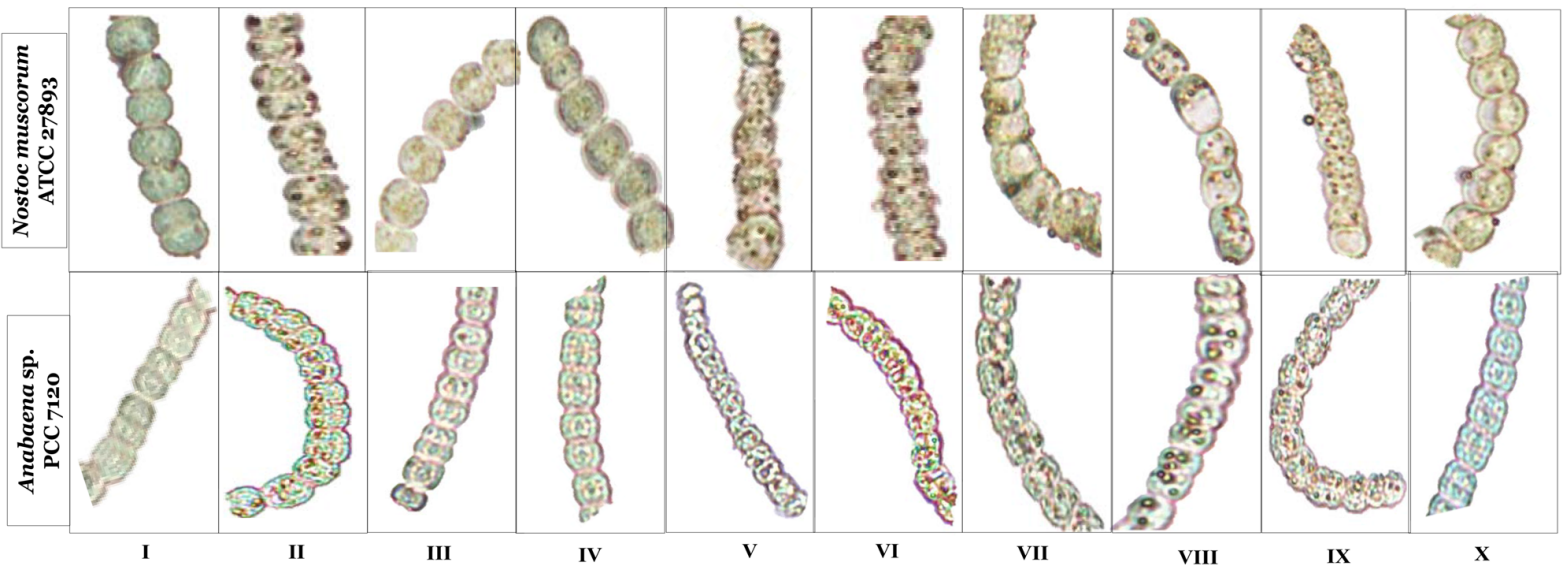
Histochemical analysis of Cd accumulation; red patches inside the cells of Cd stressed *Nostoc muscorum* ATCC 27893 and *Anabaena* sp. PCC 7120 exposed to H_2_O_2_ and NO (SNP). Where lane I: Control, lane II: Cd, lane III: Cd + H_2_O_2_, lane IV: Cd + SNP, lane V: Cd + H_2_O_2_ + PTIO, lane VI: Cd + H_2_O_2_ + _L_NAME, lane VII: Cd + SNP + NAC, lane VIII: Cd + SNP + DPI, lane IX: Cd + H_2_O_2_ + SNP + PTIO + _L_NAME, lane X: Cd + H_2_O_2_ + SNP + NAC + DPI.

Histochemical analysis of Cd inside the cells was observed in the form of red patches which were the insoluble red salt that appeared due to the complex of dithizone with Cd. Results pertaining to the in vivo visualization of Cd accumulation in both the tested organisms have been portrayed in Fig. 2. The appearance of intense red patches inside the vegetative cells of cyanobacteria showed the accumulation of Cd inside the cells. In Cd treated cells, red patches are found more intense than H_2_O_2_ and SNP treated cells. Whereas in control, no red spots have appeared. Critically intense red patches appeared in the cells under treatments of PTIO or _L_NAME even in the presence of H_2_O_2_. Treatment of SNP along with NAC or DPI; reduced the appearance of red spots inside the cell. The patches were found more intense in *Anabaena* sp. than *Nostoc muscorum* which showed that Cd easily entered into the cells of *Anabaena* sp. than *Nostoc muscorum*.

### Effect of H_2_O_2_ and NO on endogenous level of ROS and indices of damage under Cd stress

Results pertaining to the contents of SOR, H_2_O_2_ and MDA equivalents showed a sharp increase of 18, 4 and 34% respectively in *Nostoc muscorum* and 19, 7 and 37% respectively in *Anabaena* sp. (Table 1). When H_2_O_2_ and SNP were supplied to Cd stressed cyanobacteria the contents of SOR, H_2_O_2_ and MDA equivalents were declined significantly (P < 0.05). However, exposure of PTIO enhanced the level of SOR, H_2_O_2_ and MDA even in the presence of exogenous H_2_O_2_ and this increasing trend was continued when _L_NAME applied together with exogenous H_2_O_2_. While exposure of SNP along with NAC or DPI was significantly able to lessen the toxic levels of SOR, H_2_O_2_ and MDA. Furthermore, the combined addition of H_2_O_2_ and SNP along with PTIO and _L_NAME vigorously enhanced the levels of SOR, H_2_O_2_ and MDA while combined exposure of H_2_O_2_ and SNP along with NAC and DPI under the same stress declined the levels of SOR, H_2_O_2_ and MDA in both Cd stressed cyanobacteria.

**Table 1:** Effect of H_2_O_2_ and NO (SNP) on the contents of SOR, H_2_O_2_ and MDA equivalents in *Nostoc muscorum* ATCC 27893 and *Anabaena* sp. PCC 7120 exposed to Cd after 24 h of treatment.

Treatments	*Nostoc muscorum* ATCC 27893			*Anabaena* sp. PCC 7120		
	SOR	H_2_O_2_	MDA	SOR	H_2_O_2_	MDA
	[nmol (mg dry weight)^−1^]			[nmol (mg dry weight)^−1^]		
Control	3.25 ± 0.05^e^	3.57 ± 0.06^d^	0.32 ± 0.01^c^	3.57 ± 0.06^e^	4.03 ± 0.07^e^	0.41 ± 0.01^c^
Cd	3.83 ± 0.06^d^	3.71 ± 0.09^d^	0.44 ± 0.01^b^	4.24 ± 0.07^d^	4.32 ± 0.07^d^	0.55 ± 0.01^b^
Cd + H_2_O_2_	1.76 ± 0.08^ h^	2.51 ± 0.04^ h^	0.19 ± 0.00f.	2.04 ± 0.04^ h^	2.98 ± 0.09^i^	0.24 ± 0.00^f^
Cd + SNP	1.69 ± 0.02^ h^	2.46 ± 0.04^ h^	0.18 ± 0.00^f^	1.92 ± 0.05^ h^	2.85 ± 0.05^i^	0.23 ± 0.00^f^
Cd + H_2_O_2_ + PTIO	4.62 ± 0.08^b^	4.92 ± 0.09^b^	0.45 ± 0.01^ab^	5.16 ± 0.08^b^	5.65 ± 0.10^b^	0.56 ± 0.01^b^
Cd + H_2_O_2_ + _L_NAME	4.82 ± 0.08^a^	5.27 ± 0.09^a^	0.47 ± 0.01^a^	5.36 ± 0.09^a^	6.03 ± 0.10^a^	0.59 ± 0.01^a^
Cd + SNP + NAC	2.61 ± 0.07f.	3.01 ± 0.05f.	0.26 ± 0.01^de^	2.82 ± 0.05^ fg^	3.55 ± 0.06^ g^	0.32 ± 0.01^d^
Cd + SNP + DPI	2.71 ± 0.04^f^	3.22 ± 0.06^e^	0.27 ± 0.01^d^	2.91 ± 0.05^f^	3.78 ± 0.07^f^	0.33 ± 0.01^d^
Cd + H_2_O_2_ + SNP + PTIO + _L_NAME	4.12 ± 0.07^c^	4.27 ± 0.10^c^	0.46 ± 0.01^a^	4.64 ± 0.08^c^	4.87 ± 0.08^c^	0.59 ± 0.01^a^
Cd + H_2_O_2_ + SNP + NAC + DPI	2.35 ± 0.04^ g^	2.78 ± 0.05^ g^	0.25 ± 0.00^e^	2.68 ± 0.04^ g^	3.28 ± 0.06^ h^	0.29 ± 0.01^e^

Data are means ± standard error of three independent experiments with three replicates in each experiment (n = 9). Bars with different letters show significant difference at P < 0.05 significance level according to the Duncan’s multiple range test.

**Figure 3:**
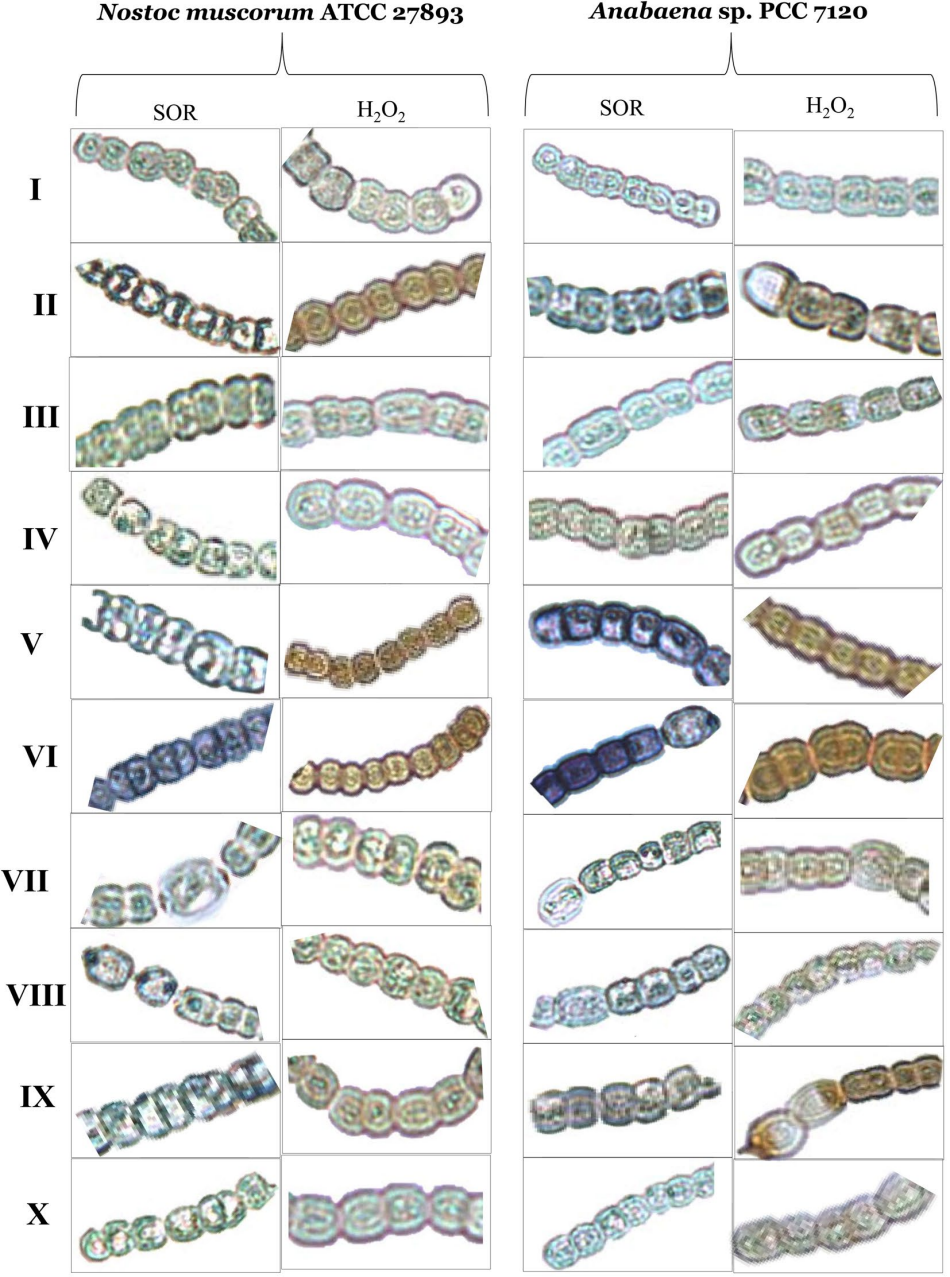
In vivo visualization of SOR; blue staining and H_2_O_2_ accumulation; brown staining inside the cells of Cd stressed *Nostoc muscorum* ATCC 27893 and *Anabaena* sp. PCC 7120 when treated with H_2_O_2_ and NO (SNP). Where lane I: Control, lane II: Cd, lane III: Cd + H_2_O_2_, lane IV: Cd + SNP, lane V: Cd + H_2_O_2_ + PTIO, lane VI: Cd + H_2_O_2_ + _L_NAME, lane VII: Cd + SNP + NAC, lane VIII: Cd + SNP + DPI, lane IX: Cd + H_2_O_2_ + SNP + PTIO + _L_NAME, lane X: Cd + H_2_O_2_ + SNP + NAC + DPI.

**Figure 4:**
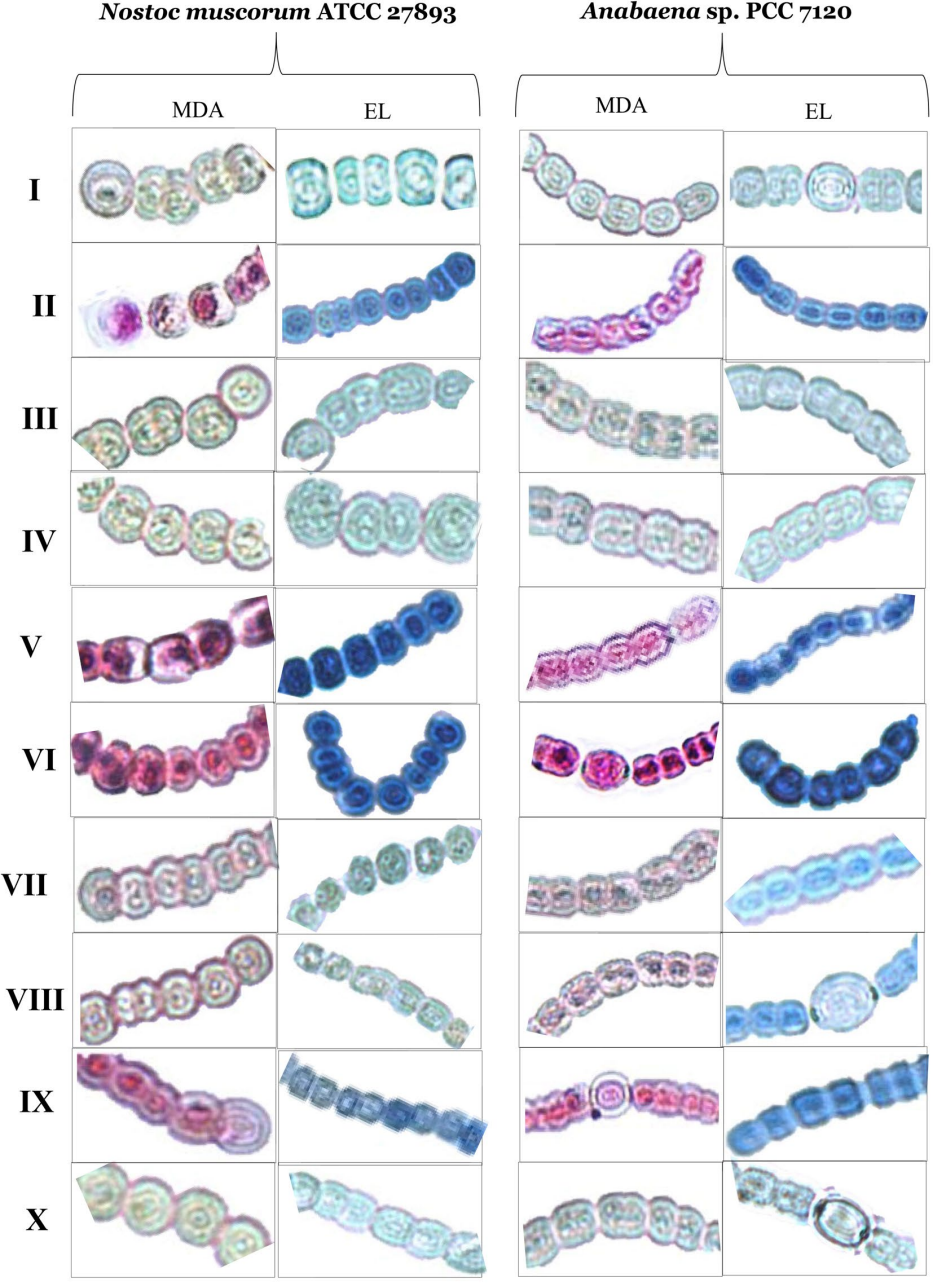
In vivo visualization of MDA; pink staining and EL; sky blue staining inside the cells of Cd stressed *Nostoc muscorum* ATCC 27893 and *Anabaena* sp. PCC 7120 when treated with H_2_O_2_ and NO (SNP). Where lane I: Control, lane II: Cd, lane III: Cd + H_2_O_2_, lane IV: Cd + SNP, lane V: Cd + H_2_O_2_ + PTIO, lane VI: Cd + H_2_O_2_ + _L_NAME, lane VII: Cd + SNP + NAC, lane VIII: Cd + SNP + DPI, lane IX: Cd + H_2_O_2_ + SNP + PTIO + _L_NAME, lane X: Cd + H_2_O_2_ + SNP + NAC + DPI.

The biochemical results of SOR, H_2_O_2_ and MDA were supported more strongly by in-vivo analysis inside the cells of both tested organisms *Anabaena* sp. and *Nostoc muscorum* (Figs. 3, 4). Blue patches were appeared by the staining with NBT for SOR, brown colored patches were appeared by DAB staining for H_2_O_2_ while pink patches were the result of staining with Shiff’s reagent for MDA, and overall effects of oxidative stress were represented as electrolyte leakage (EL) shown by the sky blue spots (Figs. 3, 4). In the figure blue, brown, pink, and sky blue patches were appeared more intense under the exposure of Cd but spots were normalized under exogenous addition of H_2_O_2_ or SNP along with Cd stress. But in the absence of NO inside the cell means under the exposure of PTIO and _L_NAME exogenously supplied H_2_O_2_ cannot limit the ROS production and intense patches were noticed. While in lack of H_2_O_2_ means under the exposure of NAC and DPI exogenously added SNP can able to limit the ROS production and faded patches have appeared inside the cells of both the organisms. This finding clearly showed the efficiency of NO towards controlling the ROS inside the cells and also clarified that the treatment of H_2_O_2_ enhanced the level of NO to normalize the endogenous level of H_2_O_2_, showed a positive relation between NO and H_2_O_2_.

### Effect of H_2_O_2_ and NO on the activity of enzymatic antioxidants under Cd stress

**Figure 5:**
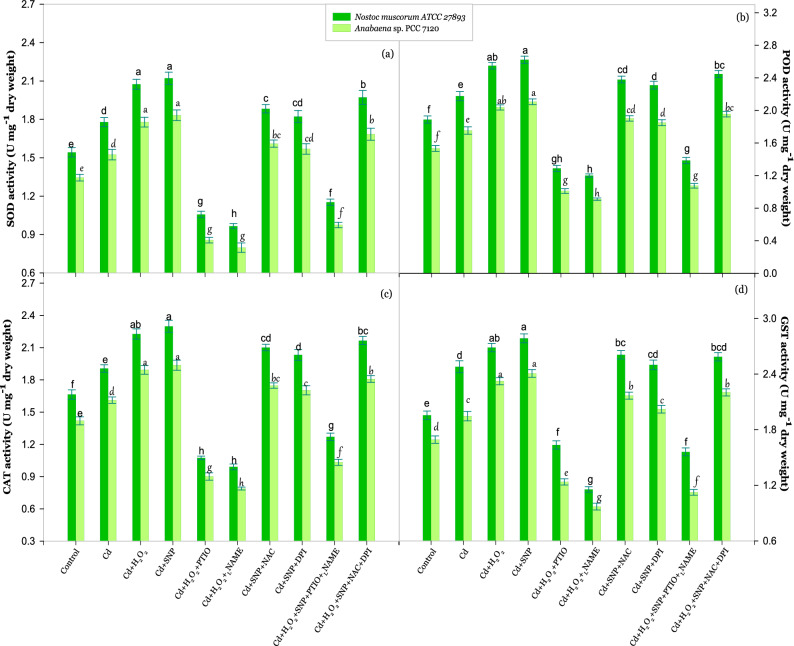
Effect of H_2_O_2_ and NO (SNP) on the activity of enzymatic antioxidants; SOD **(a)**, POD **(b)**, CAT **(c)** and GST **(d)** of *Nostoc muscorum* ATCC 27893 and *Anabaena* sp. PCC 7120 exposed to Cd after 24 h of treatment. Data presented are means ± standard error of three independent experiments with three replicates in each experiment (n = 9). Bars with different letters represent significant difference at P < 0.05 significance level according to the DMRT.

Figure 5a–d show the effect of signaling molecules on activities of enzymatic antioxidants (SOD, POD, CAT and GST) under Cd stress. Results represented that the activities of SOD, POD, CAT and GST got barely enhanced (P < 0.05) by 15, 15, 14 and 27% respectively in Cd stressed *Nostoc muscorum* and 13, 14, 14 and 20% respectively in Cd stressed *Anabaena* sp. Under similar stress activities of these antioxidants were found extremely enhanced under the exposure of H_2_O_2_ and SNP with a more pronounced effect of SNP. Contrary to this, activities of these antioxidants were got arrested under the exposure of PTIO or _L_NAME even in the presence of H_2_O_2_ while enhanced activities of these enzymatic antioxidants were found on the exposure of SNP even in the presence of NAC or DPI in both the test organisms.

**Figure 6:**
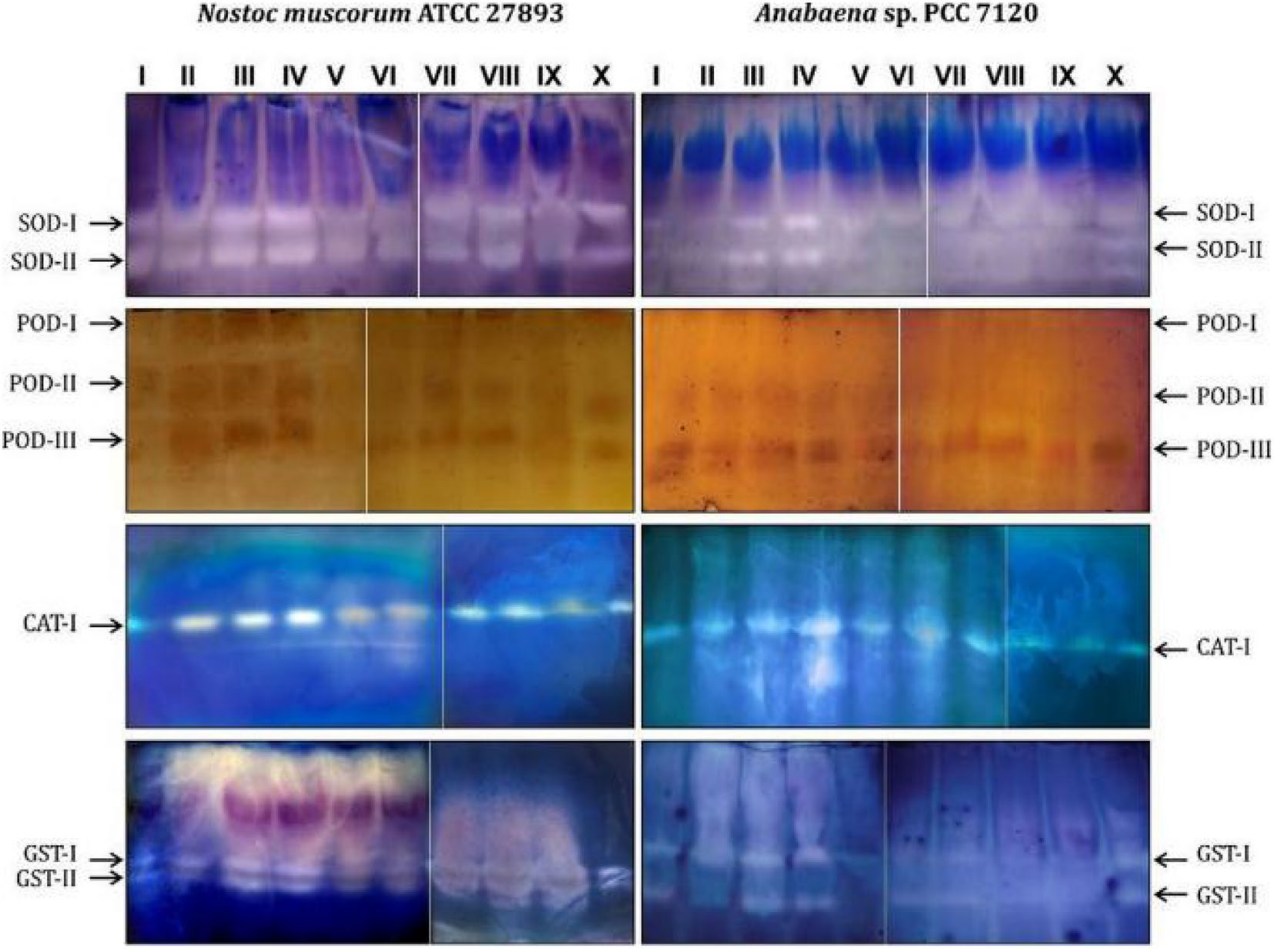
Isoenzymes profiling of SOD, POD, CAT and GST in H_2_O_2_ and NO (SNP) treated *Nostoc muscorum* ATCC 27893 and *Anabaena* sp PCC 7120 exposed to Cd stress. For the determination of isoenzyme activity, 300 μg proteins from cell extracts were loaded into the wells of native PAGE; where lane I: Control, lane II: Cd, lane III: Cd + H_2_O_2_, lane IV: Cd + SNP, lane V: Cd + H_2_O_2_ + PTIO, lane VI: Cd + H_2_O_2_ + _L_NAME, lane VII: Cd + SNP + NAC, lane VIII: Cd + SNP + DPI, lane IX: Cd + H_2_O_2_ + SNP + PTIO + _L_NAME, lane X: Cd + H_2_O_2_ + SNP + NAC + DPI; full length gels are presented in supplementary Fig. S1.

The expressions of isoenzymes of SOD, POD, CAT and GST were more strongly supported the biochemical analysis of antioxidant enzymes clearly depicted in Fig. 6. where the intensity of bands of SOD, POD, CAT and GST were found nominally intense under Cd stress and band intensity was found extremely intense under the supplementation of H_2_O_2_ and SNP with Cd. Unlikely, the bands were found negligibly appeared when NO is blocked while band slightly appeared where H_2_O_2_ is blocked and SNP is provided. In *Nostoc muscorum* the activities of antioxidants were found more intense than *Anabaena* sp. showed the resistive behavior of *Nostoc muscorum*.

Results finally clarify the signaling role of H_2_O_2_ and NO. They can detoxify the Cd stress and as it is evident form both the tested organisms by enhancing their defensive layer exopolysaccharides secretion as well as by boosting their antioxidant machinery and also by reducing the excessive level of ROS formed inside the cell. On the other hand, improvement was found more pronounced in the case of *Nostoc muscorum* showing its resistive behavior in comparison to *Anabaena* sp.

## Discussion

### H_2_O_2_ up regulates NO to enhance the growth of cyanobacteria under Cd stress

**Figure 7:**
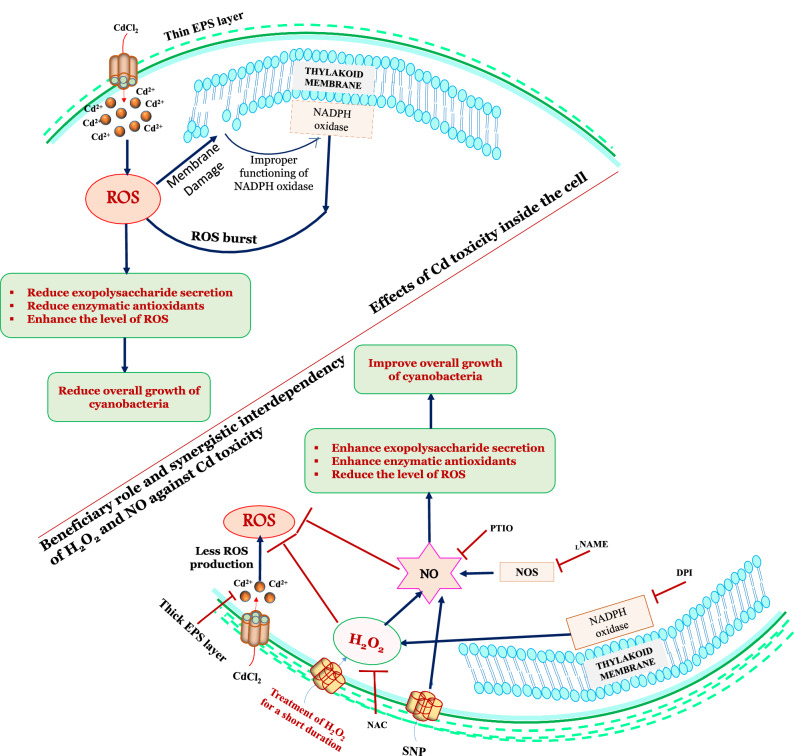
Schematic representation of negative impacts of Cd toxicity and positive synergistic mechanism of H_2_O_2_ and NO in acquisition of Cd stress tolerance in cyanobacteria.

In the present study, both H_2_O_2_ and NO successfully cope up with the Cd stress and enhance the growth of cyanobacteria which is similar to the findings of Uchida et al*.*^19^, Liu et al*.*^20^ and Nahar et al*.*^21^. The possibility of reasons behind this improved growth can be summarized into the following points. (1) H_2_O_2_ and NO can reduce the intracellular accumulation of Cd (Fig. 2) by enhancing the secretion of EPS (Fig. 1c); (2) protein content can also be enhanced by exogenous exposure of H_2_O_2_ and NO (Fig. 1d); (3) H_2_O_2_ and NO can enhance endogenous NO content (Fig. 1e) and (4) H_2_O_2_ and NO can reduce the excessive ROS contents (Table 1) by enhancing enzymatic antioxidant activities (Fig. 5a–d). Similar to this, Christou et al*.*^17^ reported that H_2_O_2_ and NO reduced the salt stress in the strawberry plants. Several studies reported that a high dose of H_2_O_2_ is used for eradication of algal bloom in ponds^22^. But against this, in the present study, a very low dose (1 µM) of H_2_O_2_ (provided for a very short time; only for 3 h) is used and very satisfactory results have been obtained against the Cd toxicity and enhanced growth of test cyanobacteria have been found under H_2_O_2_ treatment (Fig. 1a,b). Basal level of endogenous ROS and NO are very important to the cell functioning under stress condition which was proven by individual treatments of NAC, DPI, PTIO and _L_NAME along with Cd (Fig. 1b). Further experiments of the present study show the interlinked process of internal signaling of H_2_O_2_ and NO. In this way, the application of inhibitor and scavenger of NO reversed the enhanced growth of cyanobacteria by worsening the Cd toxicity inside the cells that confirms the dependency of H_2_O_2_ on NO. Our results are in consonance with the finding of Li et al*.*^16^; they revealed that under heat stress, the combined treatment of H_2_O_2_ and NO enhanced the survival percentage of maize seedlings and the stress was found worsened under the application of cPTIO. Furthermore, from the present results, it is found that NAC and DPI have not worsened the Cd toxicity in the presence of endogenous or exogenously supplied NO (because exogenously supplied SNP might fulfill the cellular level of NO that is regulated by H_2_O_2_), that showed a direct relationship between H_2_O_2_ and NO (Fig. 7) and it is cleared that H_2_O_2_ regulates NO to cope up with Cd toxicity.

### H_2_O_2_ promotes NO to enhance the EPS secretion which reduces the intracellular cd accumulation

The protective layer of EPS mainly comprises a group of biopolymers with high molecular weight which are secreted in response to any environmental stress that make a barrier against the stress^23^. Cadmium is a highly toxic metal found on Earth that disrupts the protective layer of EPS in tested cyanobacteria (Fig. 1c) which can be related with the more Cd accumulation inside the cells (i.e. displayed by red patches) demonstrated in Fig. 2. Similar to this Patel et al*.*^24^ also found the reduced EPS content along with increased As accumulation in *Nostoc muscorum* and *Anabaena* sp. Such disruptions in EPS content have been eliminated by the application of H_2_O_2_ and SNP and red patches inside the cells are less appeared (Fig. 2). However, when NO is completely arrested and H_2_O_2_ is supplied, the content of EPS has been reduced vigorously and intracellular accumulation of Cd enhanced critically. From this result, it is clear that H_2_O_2_ promotes NO to produce more EPS to chunk the intracellular metal accumulation. Similarly, Tewari et al*.*^11^ demonstrated that in *Anabaena* sp. the application of PTIO and _L_NAME reduced the EPS content and enhanced the Al accumulation inside the cells.

### H_2_O_2_ up-regulates NO to enhance the protein content of Cd stressed cyanobacteria

During adverse conditions, protein content of any organism is directly associated with the growth. In the present study, decreased protein content (Fig. 1d) subjected to Cd stress might be due to the direct impact of Cd on protein biosynthesis^25^. Contrary to this, exogenous H_2_O_2_ and NO reversed the negative impact of Cd on protein content (Fig. 1d). H_2_O_2_ and NO might be inducing the protein biosynthesis by controlling the ROS metabolism inside the cell^26^. Interestingly, ROS production was further induced (Table 1) by supplementation of PTIO and _L_NAME that resulted to the damage in protein content (Fig. 1d) which collectively reduced the growth of Cd stressed cyanobacteria.

### Exogenously supplied H_2_O_2_ and SNP promote the endogenous production of NO for balancing of ROS and electrolyte leakage induced by Cd stress

As endogenous NO is known to play an important role in all the plant developmental activities and metal stress badly reduces the NO content by interfering with nitric oxide synthase enzyme^27^. Our present study revealed that endogenous NO content was reduced under Cd stress which was further improved by treatment of H_2_O_2_ and SNP (Fig. 1e) and such increase was eliminated by adding PTIO and _L_NAME, which are in the same line with previous findings of Xuan et al*.*^28^ and Li et al*.*^16^. Elimination of NO content by _L_NAME clearly shows that nitric oxide synthase enzyme involves in NO biosynthesis inside the tested organisms. Correspondingly, Bouchard and Yamasaki^29^ have also clarified that microalgal NO is produced in a time-dependent manner under heat shock exposure which was further eliminated by adding cPTIO. Christou et al.^17^ have also demonstrated that SNP and H_2_O_2_ enhanced the NO content under NaCl stress in strawberry plants. Furthermore, exogenous SNP also dose a positive regulation of NO accumulation in the presence of NAC and DPI under Cd stress (Fig. 1e), suggesting that Cd tolerance induced by H_2_O_2_ may be achieved by increasing endogenous NO level, and NO is a downstream signal molecule of H_2_O_2_.

Moreover, Cd can block ETS (electron transport chain) or displace the iron (Fe) molecule with other protein molecules that cause the abundant production of ROS inside the cell^30^. This excessive ROS can cause direct damage to lipids, proteins, carbohydrates as well as to cellular genetic materials^31^. So the balancing of ROS inside the cell is very necessary. The current study showed that SOR, H_2_O_2_ and MDA contents were significantly increased under Cd stress (Table 1; Figs. 3, 4) which might be due to hindrance in ferredoxin pool thereby disturbance in Calvin cycle regulation^32^. Such increases in ROS were abolished by the addition of H_2_O_2_ and SNP which was in consistent with the studies of Koningshofer et al*.*^33^, Hu et al*.*^34^ and Li et al*.*^35^. A previous study also shows that at very low concentration H_2_O_2_ works as a signaling mediator and modulates deferent stress managing genes. H_2_O_2_ up-regulate the NO facilitated ABA-induced mitogen-activated protein (MAP) kinase cascade pathway to empower the defensive mechanism of maize leaves. During stress situation H_2_O_2_, can regulate NO, and NO itself works as a ROS scavenger that is the point of interaction between these two incredible signaling molecules^15^.

Our results showed that H_2_O_2_ and SNP alleviated electrolyte leakage (Fig. 4; sky blue spots) which is supported by the previous finding of Song et al*.*^36^. Similarly, Li et al*.*^16^ suggested that pretreatment of H_2_O_2_ in heat-stressed maize seedlings reduced the electrolyte leakage and endogenous MDA content to improve the overall photosynthesis and growth. Ali et al*.*^37^ also showed that the application of SNP reduced the level of H_2_O_2_ in the *Triticum aestivum* L. plant. Again the contents of these ROS species have been more pronouncedly increased under the treatment of PTIO and _L_NAME. However, in the current study, a reduced level of endogenous H_2_O_2_ content have been found under the exposure of exogenous H_2_O_2_ showed a reversible pathway in which H_2_O_2_ signals may promote endogenous NO to enhance the activity of enzymatic antioxidants that ultimately expels out the overall ROS species from the cell as demonstrated by Verma et al*.*^12^.

### H_2_O_2_ and NO enhanced the activity of enzymatic antioxidants under Cd stress

The previous study of Farooqui et al*.*^38^ in *Nostoc muscorum* disclosed that SOD, CAT and POD antioxidants were found to decrease under high concentrations of Cd. In accordance with previous studies, the current results showed that Cd reduced the level of enzymatic antioxidants (SOD, POD, CAT and GST) and bands of their isoenzymes were also found less enhanced (Figs. 5, 6). The possible reason behind this disturbance in activities of enzymatic antioxidants might be due to the alterations in basal level of ROS and thereby increased oxidative stress^27^. Cd has a strong affinity to bind with –SH groups or proteins to disturb the activity and synthesis of various enzymes^39^. In present work increased activities of SOD, POD, CAT and GST (Fig. 5a–d) were recorded on exogenous exposure of H_2_O_2_ and SNP which played a vital role in the reduction of ROS from the cells which is supported by the studies of Hasanuzzaman et al*.*^15^ and Xu et al*.*^40^. Also, the study of Qian et al*.*^41^ revealed the role of NO in positive regulation of several enzymatic antioxidants under herbicide stress in *Chlorella vulgaris*. SOD, POD, CAT and GST are known to directly catalyze the ROS scavenging reaction. A mild dose of H_2_O_2_ supplied to cyanobacteria in the present study, enhanced the activities of these enzymatic antioxidants that may regulate the AsA-GSH cycle in Cd affected cyanobacteria which is directly related to ROS scavenging process and that is why the low amount of H_2_O_2_ decreased the intracellular ROS levels^42–44^. The whole above mentioned results implied that the acquisition of Cd stress tolerance in cyanobacteria may be involved in cross-talk between H_2_O_2_ and NO and a very complex feedback regulation is found between both signaling molecules for metabolic adaptation against abiotic stress.

## Materials and methods

### Experimental organisms and culture condition

*Nostoc muscorum* ATCC 27893 and *Anabaena* sp. PCC 7120, heterocyst containing filamentous cyanobacteria were grown in BG-11 medium (pH 7.5). Their homogenous cultures were grown in controlled growth conditions; at a temp of 25 ± 2 ºC under the illumination of 75 µmol photons m^−2^ s^−1^ of photosynthetically active radiation (PAR, 400–700 nm) with a controlled regime of 14:10 h light/dark cycle.

### Experimental design and chemical treatment

Experimental design used for the present study was completely randomized design (CRD). To perform the experiments, cultures of both tested cyanobacteria were collected at their exponential phase by centrifuging them at 3000*g* for 15 min and washed with sterilized distilled water (DW). Henceforth, cyanobacterial cells were subjected to the growth medium containing concentrations of Cd (6 µM), sodium nitroprusside (SNP: a donor of NO) (10 µM), PTIO (2-phenyl-4,4,5,5-tetramethylimidazoline-1-oxyl 3-oxide); scavenger of NO (20 µM), _L_NAME (*N*ω-Nitro-l-arginine methyl ester hydrochloride); NOS enzyme’s inhibitor (100 µM), H_2_O_2_ (1 µM), NAC (*N*-acetyl-l-cysteine); scavenger of H_2_O_2_ (1 mM), DPI (diphenyleneiodonium chloride); an inhibitor of NADPH oxidase enzyme (1 µM). Accordingly, succeeding treatments were designed- Control; C (only in BG-11 medium), Cd (cadmium), Cd + H_2_O_2_, Cd + NAC, Cd + DPI, Cd + SNP, Cd + PTIO, Cd + _L_NAME, Cd + H_2_O_2_ + PTIO, Cd + H_2_O_2_ + _L_NAME, Cd + SNP + NAC, Cd + SNP + DPI, Cd + H_2_O_2_ + SNP + PTIO + _L_NAME, Cd + H_2_O_2_ + SNP + NAC + DPI.

As H_2_O_2_ is a short-lived photodegradable component so for the experiments of the present study, H_2_O_2_ was prepared very carefully in dark and added to the experimental set up at the end wherever it was required. Finally, all cultures were incubated 3 h in dark and after that they were placed for cycle 14:10 h light and dark. All the parameters were analyzed after 24 h of treatment.

### Measurement of growth

The growth of test cyanobacteria was measured in terms of dry weight. The 100 ml cultures of both the organisms were collected and centrifuged at 4000*g* for 10 min and then washed twice with distilled water. Further, pellets were dried at 80 ºC and weighed gravimetrically (Contech-CA 223, India).

### Determination of exopolysaccharides content

Exopolysaccharides (EPS) content was determined according to Sharma et al*.*^45^. For EPS extraction, 100 ml samples were taken from each treatment, centrifuged at 3000*g* for 15 min supernatants were collected and then dried separately at 40 ºC. The obtained precipitates were washed thrice with isopropanol and dried again at 37 ºC. Further, hydrolysate was analyzed for glucose by Seifter et al*.*^46^ and calculation was done by standard curve of glucose.

### Estimation of protein content

For the extraction of protein, the method of Bradford^47^ was adopted. Cells were centrifuged and homogenized with 50 mM potassium phosphate buffer (PPB) (pH 7.8) containing 1 mM EDTA and 2% polyvinyl pyrrolidone at 4 °C. Further, 0.1 ml of obtained homogenates was mixed with 2.5 ml of Coomassie brilliant blue G-250 reagent, then kept for 2 min in dark at 25 °C and absorbance was read at 595 nm. Total soluble protein content was determined by using bovine serum albumin as standard protein solution.

### Detection and quantification of NO

The NO content was determined by using Griess reagent (Sigma-Aldrich) by following the procedure of Zhou et al*.*^48^ with some modifications. For NO estimation desired volume of cultures was centrifuged and pellets were crushed in 3 ml of 50 mM cool acetic acid buffer (pH 3.6, containing 4% zinc diacetate). Further, homogenates were centrifuged and supernatant was mixed with 50 mg of charcoal. After vortex and filtration, the filtrate was leached and collected. The 1 ml of filtrate and 1 ml of Greiss reagent was mixed and incubated at room temperature for 30 min. Absorbance was taken at 540 nm. NO content was calculated by the standard curve prepared by graded solution of NaNO_2_.

### Histochemical detection of Cd

Histochemical analysis of Cd was conducted through the method of Seregin and Kozhevnikova^49^ with some minor changes. To detect the intracellular Cd accumulation; cells were collected by centrifugation then gently washed twice or thrice with double distilled water (DDW) to remove excess Cd. Obtained cells were mixed with 1 ml of dithiozone stock solution containing 6 gm of dithiozone and DW (3:1). Further 1–2 drops of acetic acid glacial were added and after 4 h of incubation; cells were observed under microscope (Leica, model- DM 2500).

### Estimation of oxidative biomarkers and indices of damage

Superoxide radical (SOR; O_2_^•-^), H_2_O_2_ contents, and lipid peroxidation were quantified by following the procedure of Elstner and Heupel^50^, Velikova et al.^51^ and Heath and Packer^52^ respectively. For SOR content the absorbance was recorded at 530 nm. The determination of SOR content was based on the formation of NO_2_ from hydroxylamine in presence of O_2_^•-^ and the content was quantified by the standard curve of nitrite (NaNO_2_). Absorbance for H_2_O_2_ was recorded at 390 nm and content was calculated by the standard curve prepared with graded solution of H_2_O_2_. SOR and H_2_O_2_ induced oxidative damage to the lipids i.e. lipid peroxidation was calculated in the form of malondialdehyde (MDA) equivalents contents. Absorbance for MDA was recorded at 532 and 600 nm and content was calculated by using an extinction coefficient of 155 mM^−1^ cm^−1^.

### In-vivo imagining of ROS (O_2_^•^¯and H_2_O_2_), membrane damage and electrolyte leakage

The Histochemical analysis of the accumulation of O_2_^•^¯and H_2_O_2_ was performed by following the method of Forster et al*.*^53^ in which cells were suspended into nitrobluetetrazolium (NBT; Sigma) and 3, 3 diaminobenzidine (DAB) for in-vivo staining respectively. MDA equivalents contents and intensity of membrane damage were visualized by using Schiff's reagent and Evan’s blue by following the methods of Pompella et al.^54^ and Yamamoto et al*.*^55^ respectively. Images were taken in a high-quality microscope (Leica, model-DM 2500).

### Enzymatic antioxidant essay

The activities of superoxide dismutase (SOD; EC 1.15.1.1), peroxidase (POD, EC 1.11.1.7), catalase (CAT; EC 1.11.3.6) and glutathione-S-transferase (GST, EC 2.5.1.18) were analyzed by following the methods of Giannopolitis and Ries^56^, Gahagan et al*.*^57^, Aebi^58^ and Habig et al*.*^59^ respectively. For SOD activity, the photoinhibition of NBT was recorded at 560 nm after 20 min illumination of light (100 µmol photons m^−2^ s^−1^). One unit of SOD activity is demarcated as the required amount of enzyme to cause 50% inhibition in the reduction of NBT. For POD, absorbance of the reaction mixture was recorded at 430 nm for 3 min. The activity of the enzyme was calculated by using an extinction coefficient of 25.5 mM^−1^ cm^−1^ and one unit of enzyme activity is defined as 1 nmol pyrogallol oxidized min^−1^. Activity of CAT was determined by monitoring the decrease in absorbance at 240 nm in the reaction mixture^58^. The activity of enzyme was calculated by using an extinction coefficient of 39.4 mM^−1^ cm^−1^. Here 1 nmol H_2_O_2_ dissociated min^−1^ was equivalent to one unit of enzyme activity. For GST, absorbance of reaction mixture was recorded at 340 nm. Enzyme activity was calculated by an extinction coefficient of 9.6 mM^−1^ cm^−1^ and one unit of enzyme activity is equivalent to 1 nmol of CDNB conjugates formed min^−1^.

### Native polyacrylamide gel electrophoresis for isoenzyme profiling

Native–PAGE analysis was carried out on discontinuous polyacrylamide gels (PAGE) with 4.5% polyacrylamide in stacking and with its varying concentrations (10% for SOD and GST, 6% for CAT and 8% for POD). The separation of individual isoenzyme was performed in a vertical gel electrophoretic unit (GeNei, India) by considering the method of Laemmli^60^. A uniform amount (300 mg) of proteins mixed with sample buffer (0.5 M Tris–HCl, pH 6.8) was loaded in each well and proteins were electrophoretically separated at 80 V through stacking gel followed by 120 V in the separating gel at 4 ºC. For visualizing SOD isoenzymes, the gels were immersed in PPB (50 mM; pH 7.8) containing NBT (1.125 mM) in darkness for 20 min and followed by incubation in PPB containing TEMED (28 mM) and riboflavin (28 mM) in dark for 15 min. The gels were then placed in PPB containing mM EDTA (0.1 ml) and exposed to light for 20 min at 25 ºC^61^. For POD, gels were immersed in 1 mg ml^−1^ benzidine and 1 mM H_2_O_2_ in 0.1 M Tris–acetate buffer (pH 5.0) at 25 ºC till the brown colored bands appeared^62^. For CAT isoenzymes gels were incubated with H_2_O_2_ (0.01%) for 15 min, rinsed with water, and shaken in freshly prepared solutions each of 0.1% FeCl_3_ and K_3_Fe(CN)_6_ at 25 ºC until the achromatic bands appeared^63^. For GST isoenzyme staining a reaction mixture containing 0.1 M PPB (pH 6.5), GSH (5.0 mM), CDNB (1.0 mM) and NBT (1 mM) was used and bands were developed by illuminating the gel under 25 µmol photon m^−2^ s^−1^ of photon flux density^64^. Photographs of isoenzymes were captured with digital camera, photo-plate was prepared in CorelDRAW and image quality was enhanced in Photoshop 7.0.

### Statistical analysis

Statistical analysis of variance (ANOVA) was performed to test the significance at probability level P < 0.05. Duncan’s multiple range test was applied to compare significant differences among the mean values. Graphical representations are the means of three independent experiments with three replicates in each experiment (n = 9). Lower case letter (a, b, c, d, e, f, g, h, i, j) shows statistical significance.

## Conclusions

From the aforementioned results, it can be concluded that there is an interlinked pathway between NO and H_2_O_2_ that exists. In Fig. 7, it is clear that Cd create oxidative stress inside the cell and produces more ROS and creates membrane damage by increasing MDA equivalents contents^15,21,65^. But exogenously supplied NO and H_2_O_2_ promote the EPS secretion and check the entrance of Cd inside the cells after that they also enhance the antioxidant system and endogenous NO content that's why the level of ROS get minimized. Here, the low amount of exogenous H_2_O_2_ works as a signal transmitter to enhance the beneficial amount of RNS species that indirectly respond to the balancing of antioxidants to cope up with cd stress. Afterward PTIO and _L_NAME with H_2_O_2_ block the internal NO that creates a disturbance in the working of signaling mechanism of H_2_O_2_ and NO here it is clear that exogenously supplied H_2_O_2_ cannot work without NO. However, application of NAC and DPI cannot disturb the signaling of NO. Hence, from this it is clear that NO is a down-stream regulator of H_2_O_2_ but the pathway of signaling between both the molecules is very complex because of their multidimensional roles in a stressful environment.

## Supplementary Information

Supplementary Figure S1.
