# Supplementary material for: Regulation of redox homeostasis in cadmium stressed rice field cyanobacteria by exogenous hydrogen peroxide and nitric oxide
Source: Sci Rep. 2021 Feb 3;11:2893. doi: 10.1038/s41598-021-82397-9 (PMC7858583; doi:10.1038/s41598-021-82397-9)
Supplement: Supplementary file 1 — Supplementary Figure S1. [file 41598_2021_82397_MOESM1_ESM.pdf]

Title: Regulation of redox homeostasis in cadmium stressed rice field cyanobacteria by exogenous hydrogen peroxide and nitric oxide

Authors: Nidhi Verma and Sheo Mohan Prasad

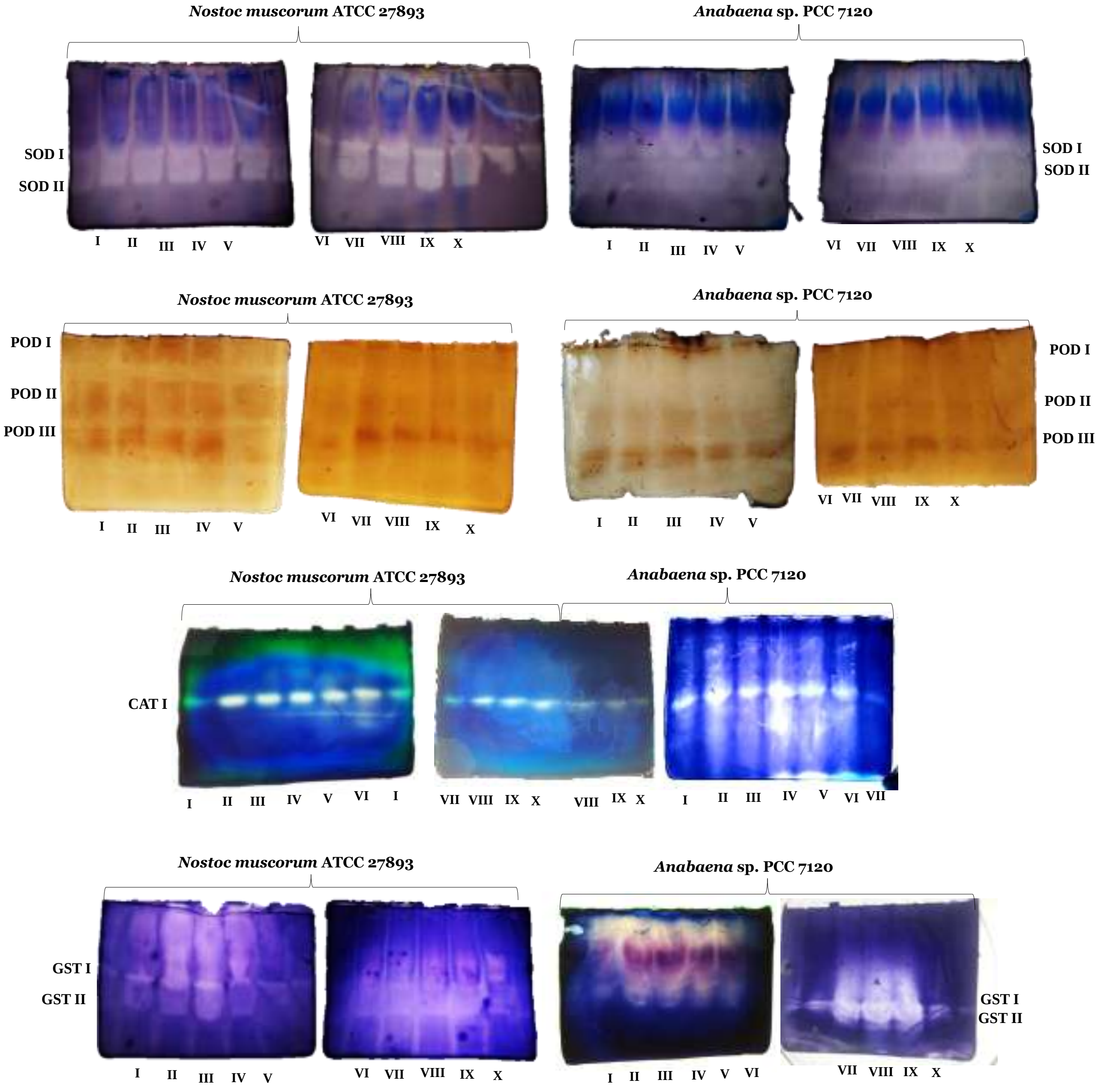

Figure. S1. Full length gel images of isoenzymes profiling of SOD, POD, CAT and GST in H_2O_2 and SNP treated *Nostoc muscorum* ATCC 27893 and *Anabaena* sp PCC 7120 exposed to Cd stress. For the determination of isoenzyme activity, 300 µg proteins from cell extracts were loaded into the wells of native PAGE; where **lane I**: Control, **lane II**: Cd, **lane III:** Cd+H_2O_2, **lane IV:** Cd+SNP, **lane V:** Cd+H_2O_2+PTIO, **lane VI:** Cd+H_2O_2+$_L$NAME, **lane VII:** Cd+SNP+NAC, **lane VIII:** Cd+SNP+DPI, **lane IX:** Cd+H_2O_2+SNP+PTIO+$_L$NAME, **lane X:** Cd+H_2O_2+SNP+NAC+DPI.
